# X-optogenetic inhibition of VTA GABA neurons for wireless deep-brain stimulation and suppression of scratching behavior in freely moving mice

**DOI:** 10.1016/j.mtbio.2026.103137

**Published:** 2026-04-17

**Authors:** Bin Lan, Haiying Liu, Jinwei Xu, Yifan Zhang, Dongyan Li, Peng Gao, Wenli Zhang, Wenting Wang, Galong Li, Hongbing Lu

**Affiliations:** a School of Biomedical Engineering, Air Force Medical University, Xi'an, China; b Department of Neurobiology, School of Basic Medicine, Air Force Medical University, Xi'an, China; c Institute for Chinese Medicine Frontier Interdisciplinary Science and Technology, Shaanxi University of Chinese Medicine, Xixian New Area, Shaanxi Province, 712046, China

**Keywords:** X-ray, Scintillator, Optogenetics, Opsins, Neuromodulation

## Abstract

X-ray-mediated wireless optogenetic neurostimulation (X-optogenetics) at any brain depth is a powerful technology for neuroscience studies and neurological disorder treatment. However, there is still a lack of effective and safe X-optogenetic neural inhibition technology because of the need for a precise spectral match degree between scintillator and inhibitory opsins and sufficient light intensity emitted from X-ray-induced scintillation. Here, we report an efficient X-optogenetic neural inhibition technology that achieves both wireless deep-brain stimulation and rapid behavioral suppression in freely moving mice. First, we developed two novel inorganic scintillators: terbium-doped gadolinium oxysulfide (Gd_2_O_2_S: Tb) and europium-doped yttrium oxide (Y_2_O_3_: Eu). The Gd_2_O_2_S: Tb and Y_2_O_3_: Eu can absorb X-ray energy and emit green and red visible light, respectively. We demonstrated that the visible light emitted from X-ray-mediated scintillation effectively activated two inhibitory opsins (ArchT and eNpHR 3.0), which facilitated successful inhibition of the GABAergic neural activity in the ventral tegmental area (VTA) of mice. Further, this X-optogenetic neural inhibition technology wirelessly and significantly suppressed itch-induced scratching bouts (about 52%) in awake, freely moving mice. Importantly, owing to the superior radioluminescence performances of scintillators, our X-optogenetic neural inhibition technology only required a low dose of X-ray (2.78 Gy), which is substantially lower than that (7 Gy) reported in a previous study. By enabling wirelessly efficient and safe neural inhibition in the deep brain, this X-optogenetic technology holds promise for advancing neuroscience investigations and therapeutic applications in large living subjects.

## Graphical abstract

**Figure:**
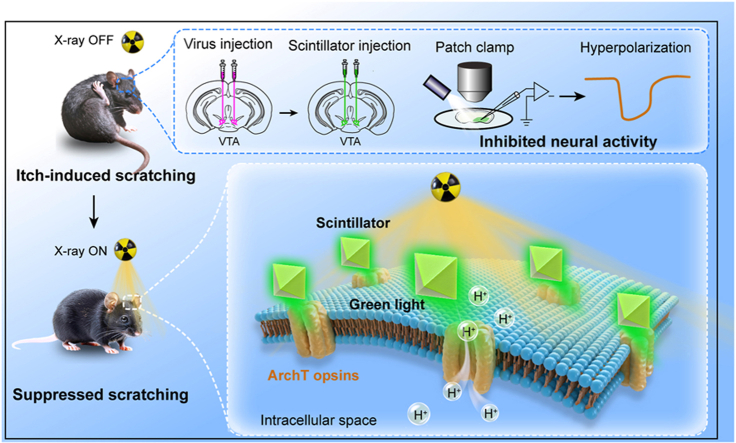

## 1 Introduction

Optogenetics is a revolutionary technology in neuroscience due to unprecedented spatiotemporal resolution [[1], [2], [3], [4], [5]]. It employs visible light to precisely activate light-sensitive opsins and modulate activities of genetically engineered neurons [[6], [7], [8], [9]]. Recently, optogenetics opened new pathways for clinical therapeutic applications [[10], [11], [12], [13], [14], [15]]. Classic optogenetic technologies rely on invasive implanted rigid optical devices (such as fiber and diodes), which were used to deliver high-power visible light into target regions in the brain and overcome light scattering and absorption in biological tissue [[16], [17], [18], [19]]. Notably, this technology is limited for the scope of *in vivo* experiments since the animal is tethered to an optical fiber and restricted around the light source, raising possible risks including physical tissue damage, phototoxicity, and thermal effects [[20], [21], [22], [23], [24], [25], [26]]. To address the above-mentioned limitations, wireless optogenetic technologies have been developed [27,28] using transducers that absorb external energy and emit visible light within the brain for neuromodulation. For example, near-infrared light (NIR) mediated wireless optogenetics has attracted great attention by leveraging upconversion nanoparticles to precisely modulate neuronal activity [[29], [30], [31], [32]]. Great efforts have been made to improve the efficacy of deep-brain wireless stimulation, including developing high-performance luminescence nanoparticles and increasing penetration depth of NIR light through the skull and brain tissue [33,34]. Recently reported broadband NIR phosphors (such as Cr^3+^-activated Ca_2_GdZrSnGa_3_O_12_ and Zn_1−x_Mg_x_Al_2_O_4_) exhibited superior performances that simultaneously deliver red-shifted emission, high quantum efficiency, and thermal robustness, showing great potential for non-invasive neurostimulation and clinical therapeutic applications [35,36]. Other external energies, such as magnetic field [37,38] and ultrasound [39,40], have also been explored but are hindered by poor temporal resolution, energy attenuation, and restricted moving area during free-behavior studies. Therefore, there is an urgent need for an alternative energy source with superior tissue-penetrating capability to enable efficient wireless optogenetics.

X-optogenetics that employs X-ray and injectable micro/nano scale scintillators to emit visible light has emerged as a compelling solution for wireless optogenetics [41,42]. It is because X-ray has irreplaceable advantages, including excellent collimation and controllability, negligible energy absorption in biological tissues, and minimal thermal effect [43,44]. In addition, X-ray is considered a potential external energy for deep-brain stimulation in large animals. Implementing effective X-optogenetics requires careful consideration of multiple factors. First, the scintillator's emission spectrum matches well with the opsin's absorption profile. Second, scintillators show high luminescent efficiency under X-ray irradiation. Third, both scintillators and X-rays are safe during neurostimulation and behavioral control. In recent years, the feasibility of X-optogenetic neural activation has been verified, including enhancing cellular and synaptic function [41], activating opsin-expressing cortical neurons [42], producing neuronal depolarization [45], inducing position preference behavior in mice [46], and using various scintillators, including cerium-doped lutetium oxyorthosilicate (LSO: Ce), gadolinium tungstate nanoparticles (Gd_2_(WO_4_)_3_: Eu), and Ce-doped Gd_3_(Al, Ga)_5_O_12_ (Ce: GAGG). However, X-optogenetic neural inhibition technology has been rarely reported, which is conducive to investigating neural mechanism and treat neurological disorders with excessive neural activity (such as epilepsy, Parkinson's disease, and Alzheimer's disease) [[47], [48], [49], [50], [51], [52], [53], [54], [55], [56]]. Until now, only one work reported an X-optogenetic neural inhibition technology that relies on high-dose X-ray exposure (up to 7 Gy) [46], raising safety concerns. Notably, X-optogenetic neural inhibition is indeed more difficult than activation, due to the need for more precise spectral match degree and sufficient light intensity, significant spectral interference, and low expression efficiency of inhibitory opsins on neurons. To develop effective and wireless X-optogenetic technology, scientists have been committed to synthesize high-performance scintillators that match well with the activation spectrum of inhibitory opsin and reduce the X-ray dose, but the progress has been slow.

**Scheme 1:**
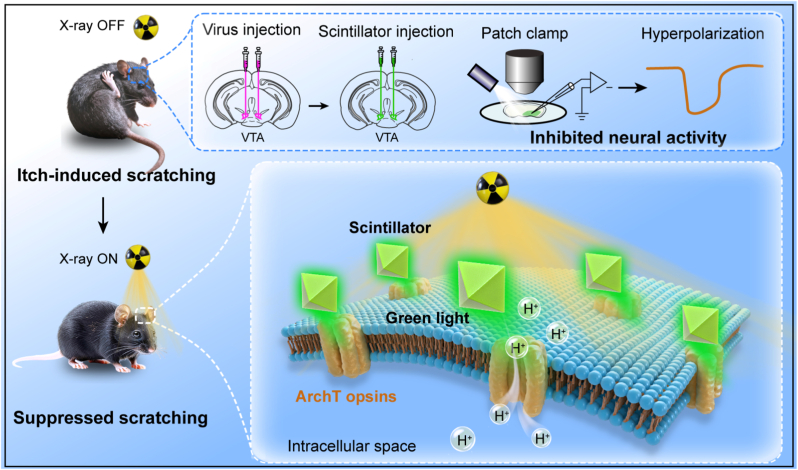
Wireless X-optogenetic neural inhibition and scratching behavior suppression in a mouse model of acute neck itch by radio luminescent scintillators under X-ray irradiation. The scintillators act as energy transducers, absorbing X-ray photons and subsequently emitting green light with wavelengths matching the activation spectra of inhibitory ArchT opsins. Upon exposure to the emitted green light, ArchT expressed on neurons of the VTA opens and triggers the hydrogen ion (H^+^) efflux, resulting in hyperpolarization and subsequent inhibition of neuronal activity. The X-optogenetic neural inhibition effectively suppressed scratching behavior in a mouse model of acute itch.

Here, we report an efficient and safe X-optogenetic neural inhibition technology using highly luminescent scintillators under low-dose X-ray exposure (Scheme 1). In specific, the scintillators induced pronounced membrane hyperpolarization in electrophysiological experiments. For *in vivo* experiments, the scintillators achieved remote deep-brain stimulation in ventral tegmental area (VTA) and successful scratching behavioral suppression (about 52%) in acute neck itch and freely moving mice. The efficient and safe X-optogenetic neural inhibition technology has the potential for future neuroscience investigation and neurological disorder treatment.

## 2 Experimental section

### 2.1 Animals

Adult male C57BL/6J inbred mice (purchased from SPF (Beijing) Biotechnology Co., Ltd) were used in all experiments. All mice were group-housed with food and water ad libitum under controlled conditions (humidity: 40%-60%; temperature: 22 - 25 °C) with a 12-h light/dark cycle. All experimental procedures were approved by the Institutional Animal Care and Use Committee of the Air Force Medical University, and animals’ care and use strictly adhered to institutional guidelines and governmental regulations.

### 2.2 Viral vectors

The viruses were used as follow: rAAV-VGATI-ArchT-EGFP (Serotype 2/9, titer 5.11 × 10^12^ copies/mL), rAAV-VGATI-EGFP (Serotype 2/9, titer 5.01 × 10^12^ copies/mL), rAAV-VGATI-eNpHR 3.0-mCherry (Serotype 2/9, titer 5.08 × 10^12^ copies/mL), and rAAV-VGATI-mCherry (Serotype 2/9, titer 5.08 × 10^12^ copies/mL) were obtained from BrainVTA (Wuhan) Co. Ltd.

### 2.3 Stereotaxic surgery

Mice were anesthetized with 1% pentobarbital (100 mg/kg) and secured in a stereotaxic device (RWD Life Science Inc., Shenzhen, China) using ear bars and a nose clamp. Ophthalmic ointment was used to prevent corneal drying. Craniotomies were performed using a micro-drill after a midline scalp incision to expose the skull. Viral vectors or scintillator solutions were injected using a 10 μL microsyringe equipped with a glass pipette (the tip diameter of 15-25 μm) via a microinjection pump. According to the Paxinos and Franklin Mouse Brain Atlas (2nd edition), stereotaxic coordinates relative to bregma for intracerebral injections were as follows: anteroposterior (AP): −3.2 mm; mediolateral (ML): ±0.5 mm; dorsoventral (DV): −4.15 mm. All injections were performed bilaterally at a rate of 60 nL/min, delivering either 400 nL of viral vectors or 600 nL of scintillator solution per side. The micropipette was left in place for an additional 10 min before withdrawal. For scintillator injections, the scintillator was dissolved in physiological saline to prepare a 50 mg/mL solution. This solution was subjected to sonication for at least 30 min to inhibit particle aggregation.

### 2.4 Scintillator biocompatibility assay i*n vitro*

HEK293 cells were seeded in a 96-well plate and incubated with culture media containing different concentrations of the scintillator (0, 1, 5, 10, 20, 30, 40, and 50 μg/mL) for 24 h. Following the incubation, CCK-8 reagent was added to each well at a 1:9 ratio (reagent: medium). The plate was then incubated for an additional 1 h. The absorbance at a wavelength of 450 nm was recorded using a multi-mode microplate reader to determine cell viability.

### 2.5 Preparation of scintillator-embedded thin films

Scintillating particles cannot maintain a stable concentration in a solution that was used in an electrophysiological experiment because they would be swept away by the flow or form local precipitation. Thus, scintillators need to be immobilized within a transparent polymethylpropanamide (PMMA) film. For preparation, take 4 mL of chloroform and dissolve 0.4 g of PMMA powder in it, then add 1 mg of scintillator powder and disperse the mixture thoroughly using an ultrasonic cleaner. Drop the solution onto a glass coverslip to form a solid film, allowing the chloroform solvent to fully evaporate. Peel off the film and cut it into the proper size with scissors.

### 2.6 Slice electrophysiology

Whole-cell patch clamp recordings were performed using acute brain slices of mice that had received stereotaxic viral injections in VTA. Before preparing slices, these mice were anesthetized with isoflurane and transcardially perfused with ice-cold carbogenated cutting solution (containing 115 mM choline chloride, 2.5 mM KCl, 1.25 mM NaH_2_PO_4_, 0.5 mM CaCl_2_, 8 mM MgCl_2_, 26 mM NaHCO_3_, 10 mM D-(+)-glucose, 0.1 mM ascorbic acid, and 0.4 mM sodium pyruvate) at conditions (95% O_2_, 5% CO_2_, pH 7.4, 295-300 mOsm/L). Following rapid brain extraction, 300 μm of coronal slices containing VTA regions were sectioned by a vibratome (VT1200S, Leica Microsystems). The slices were incubated in oxygenated cutting solution (32 °C) for 30 min in a holding chamber, then transferred to artificial cerebrospinal fluid (pH 7.4, 295-300 mOsm/L) that contains 119 mM NaCl, 2.3 mM KCl, 1.0 mM NaH_2_PO_4_, 26 mM NaHCO_3_, 11 mM D-(+)-glucose, 1.3 mM MgCl_2_, and 2.5 mM CaCl_2_. After 1 h recovery at room temperature, slices were transferred to a recording chamber and continuously perfused with oxygenated ACSF (3 mL/min) at 28 °C.

Neurons were observed through infrared differential interference contrast (IR-DIC) microscopy (Olympus BX51). Under an appropriate filter set, GABA neurons were identified by fluorescence imaging. Whole-cell voltage-clamp recordings were performed using borosilicate pipettes (3-5 MΩ) back-filled with internal solution containing 10 mM Na-phosphocreatine, 128 mM K-gluconate, 1.1 mM EGTA,10 mM HEPES, 5 mM Mg-ATP, and 0.4 mM Na-GTP. After amplification via a MultiClamp 700B amplifier, signals were digitized using a Digidata 1440A interface (Molecular Devices). After determining the resting membrane potential, a scintillator-embedded polymer film was positioned 1 mm above the slice. A UV light source was fixed 5 cm from the film for photoactivation. Recordings were conducted using a microelectrode amplifier with bridge and voltage-clamp mode of operation (MultiClamp 700B) filtered at 5 kHz and sampled at 20 kHz (Digidata 1500B). Data were further analyzed by Clampex 10.7 software (Molecular Devices).

### 2.7 Neck models of acute itch

As previously described, the acute itch behavior tests of chloroquine-treated mice were conducted with modifications. Before the experiment, the hair on the nape of the mouse's neck was removed. Mice were individually placed in opaque plastic cages with fresh bedding and allowed to acclimate for 30 min. Then, 20 μL chloroquine (10 μg/μL, Sigma-Aldrich) was injected into the nape of the mouse's neck. Video recording commenced for 30 min immediately following the onset of the first scratching bout. During this period, the mice received either ray stimulation or no stimulation, according to the experimental paradigm, in the absence of an observer. The recorded videos were later replayed in slow motion, and scratching bouts were quantified by visual inspection. A scratching bout was defined as a discrete episode in which the mouse scratched the injection site by lifting its hind paw and then returning the paw to the ground or to its mouth.

### 2.8 X-optogenetics behavioral test

During behavioral testing, stimulation was induced continuously at 150 kV and 0.5 mA, within the 3-min X-ray-ON period following a 3-min X-ray-OFF period, repeated 5 times over the total period of 30 min after the animal's first scratching behavior. Scratching bouts were subsequently quantified by reviewing the video recordings, separately for the non-stimulation and stimulation periods. For the control group (opsin +, scintillators +, X-ray -), the entire session was conducted without X-ray exposure. During the quantification of scratching bouts, the same temporal segmentation (i.e., the alternating 3-min intervals) as applied to the stimulated groups was used for analysis.

### 2.9 Histology and fluorescent immunostaining

After anesthesia with 1% sodium pentobarbital, the animals underwent cardiac perfusion with PBS (20 mL, 0.01 M), followed by perfusion with ice-cold paraformaldehyde (PFA, 4%) in phosphate-buffered saline (PBS). After being fixed with 4% PFA, the mouse brain was extracted 5 min later. Subsequently, the brain was placed in a sucrose solution (30%) for 3 days. To evaluate c-Fos expression in the VTA, the dissection process was performed 1.5 h after the start of video recording. For brain sectioning, the tissue was embedded in OCT compound, frozen, and then cut into thick sections (50 μm) by a cryostat (Leica, CM1950S). Brain slices containing the VTA regions of interest were selected and washed with PBS. A blocking solution was prepared (100 mL of 0.01 M PBS, 0.3 mL Triton X-100, and 0.5 g BSA powder), and the brain slices were incubated in an adequate amount of this blocking solution for 1 h. The slices were then transferred into a c-Fos rabbit anti-antibody solution (1:1000 dilution) for 16 h, followed by PBS washes. The mouse brain slices were subsequently incubated in a donkey anti-rabbit secondary antibody solution (1:500 dilution) for 4 h (the secondary antibody was Alexa Fluor 488-labeled for the ArchT-Gd_2_O_2_S: Tb group experiment and Alexa Fluor 594-labeled for the eNpHR 3.0-Y_2_O_3_: Eu group experiment). The slices after washing again with PBS were incubated in a Hoechst solution (1:1500 dilution) at room temperature for 10 min, washed, and mounted onto glass slides for subsequent fluorescence confocal imaging.

For c-Fos quantification analysis, c-Fos signals within the brain region were automatically counted using ImageJ software. The imaging parameters were kept consistent for all sections, and the counting experiment was conducted in a blinded manner.

### 2.10 Data analysis

No statistical methods were used to pre-determine sample sizes, but our sample sizes are similar to those reported in previous work and are typical of the field. All data are presented as mean ± SEM. Statistical analyses were made through Prism 10.0 (GraphPad Software). Normality and Homogeneity of variances were evaluated using the Shapiro–Wilk test and the Brown–Forsythe test, respectively. For specific analyses, one-way ANOVA followed by Tukey's test and two-way ANOVA followed by Tukey's multiple comparisons test were applied. Non-normally distributed data were assayed using nonparametric tests. A P value < 0.05 was considered statistically significant, and exact P values are displayed in the figures.

## 3 Results and discussion

### 3.1 Superior luminescence properties of Gd_2_O_2_S: Tb and Y_2_O_3_: Eu scintillators

We prepared two high-performance scintillators (Gd_2_O_2_S: Tb and Y_2_O_3_: Eu), which show superior radioluminescence under X-ray exposure. The emitted light from these scintillators can be used to activate the commonly used inhibitory optogenetic opsins (ArchT and eNpHR 3.0) for silencing the neuronal firing. ArchT is an outward proton pump derived from *Halorubrum* strain TP009 [57], while eNpHR 3.0 is an optimized version of chloride pump originated from *Natronomonas pharaonis* [58]. Both ArchT and eNpHR 3.0 can be activated by visible light with wavelengths ranging from 450 nm to 650 nm [[57], [58], [59]]. In this work, we aim to investigate the feasibility of the Gd_2_O_2_S: Tb and Y_2_O_3_: Eu scintillators for activating the inhibitory opsins ArchT and eNpHR 3.0, respectively, and for developing wireless X-optogenetic neural inhibition technology.

**Fig. 1:**
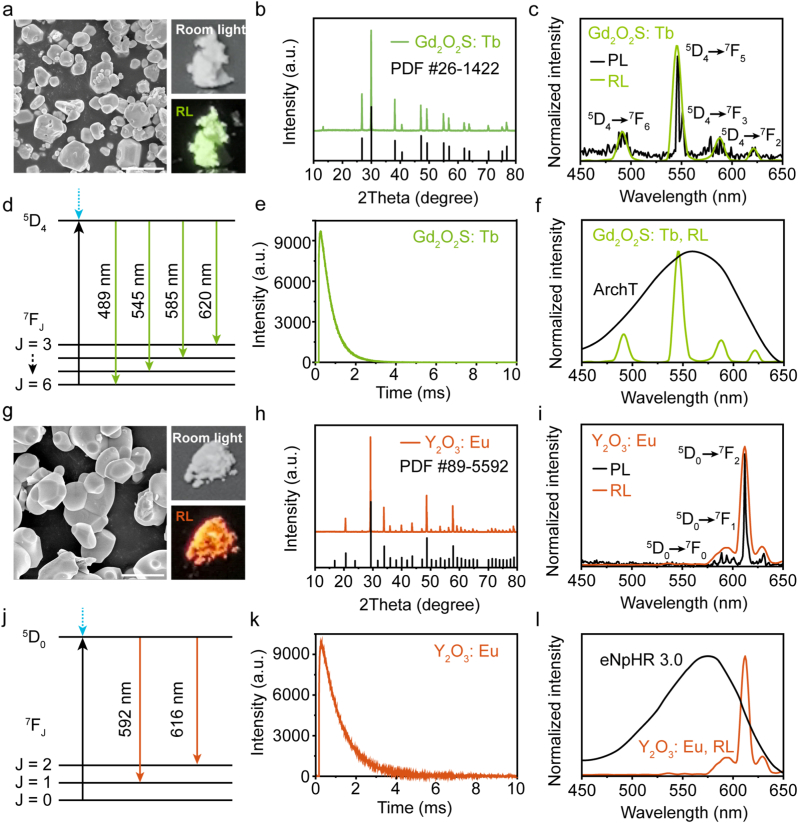
**Characterization of Gd_2_O_2_S: Tb and Y_2_O_3_: Eu scintillators. (a)** SEM of Gd_2_O_2_S: Tb scintillators. Photographs of Gd_2_O_2_S: Tb powder under room light and X-ray-induced (150 kV, 0.5 mA) radioluminescence (RL). Scale bars: 5 μm. **(b)** XRD pattern of Gd_2_O_2_S: Tb. **(c)** Emission spectra of Gd_2_O_2_S: Tb under UV excitation (photoluminescence, PL) and X-ray excitation (RL). **(d)** Schematic illustration of the RL mechanism in Gd_2_O_2_S: Tb. **(e)** Transient spectra of Gd_2_O_2_S: Tb. **(f)** RL spectrum of Gd_2_O_2_S: Tb matches with the activation spectrum of ArchT. **(g)** SEM of Y_2_O_3_: Eu scintillators. Photographs of Y_2_O_3_: Eu powder under room light and X-ray-induced (150 kV, 0.5 mA) RL. Scale bars: 5 μm. **(h)** XRD pattern of Y_2_O_3_: Eu. **(i)** PL and RL spectra of Y_2_O_3_: Eu. **(j)** Schematic illustration of the RL mechanism in Y_2_O_3_: Eu. **(k)** Transient spectra of Y_2_O_3_: Eu. **(i)** RL spectrum of Y_2_O_3_: Eu matches with the activation spectrum of eNpHR 3.0.

Scanning electron microscopy (SEM) images revealed that Gd_2_O_2_S: Tb scintillators exhibit a block-like cubic morphology with an average diameter of 1.31 ± 0.68 μm (Fig. 1a, Fig. S1a). As the HR-TEM image displayed in Fig. S1b, the lattice spacing of 3.10 Å is well matched with the (111) crystal plane of the Gd_2_O_2_S: Tb scintillator. X-ray diffraction (XRD) spectra confirmed the crystalline phase of Gd_2_O_2_S (Fig. 1b), showing good quality without any impure peaks. Energy dispersive spectrometer (EDS) analysis demonstrated the uniform distribution of Gd, O, S, and Tb (Fig. S2a and Table S1). The ultraviolet (UV) induced photoluminescence (PL) and X-ray induced radioluminescence (RL) of scintillators were further studied. Gd_2_O_2_S: Tb emit green light under UV or X-ray irradiation (Fig. 1c). And the Gd_2_O_2_S: Tb exhibited an ultrahigh light yield (60000 photons/MeV), indicating that the scintillator is a good X-ray energy transducer [60,61]. PL/RL spectra of Gd_2_O_2_S: Tb exhibited four narrow bands in the visible range, with peaks located at 491 nm, 545 nm, 588 nm and 621 nm, corresponding to the radiative transitions of Tb^3+^ ions (from ^5^D_4_ to ^7^F_6_, ^7^F_5_, ^7^F_4,_ and ^7^F_3_), as shown in Fig. 1c and d. The most intense green emission centered at 545 nm of Gd_2_O_2_S: Tb matches well with the activation spectrum of ArchT (Fig. 1f).

In contrast, the Y_2_O_3_: Eu scintillators showed a similar block-like cubic shape with an average particle size of 3.38 ± 1.31 μm (Fig. 1g, Fig. S1c). As the HRTEM shown in Fig. S1d, the interplanar distance between the adjacent lattice planes was estimated to be about 3.34 Å, which corresponds to the (222) crystal plane of the body-centered cubic Y_2_O_3_ phase. The clearer lattice fringes in the HRTEM images show that Y_2_O_3_: Eu exhibit good crystallinity. XRD spectra confirmed the crystalline phase of Y_2_O_3_ (Fig. 1h). EDS confirmed the uniform element distribution of Y, O, and Eu (Fig. S2b, Table S2). Under UV (or X-ray) irradiation, Y_2_O_3_: Eu emits orange-red light (Fig. 1i). Red emission with maximum wavelength at 612 nm of Y_2_O_3_: Eu is attributed to the radiative transition of Eu^3+^ ions (from ^5^D_0_ to ^7^F_2_) (Fig. 1j), indicating the Y_2_O_3_: Eu is suitable for activating red-shifted eNpHR 3.0 (Fig. 1l).

Both Gd_2_O_2_S: Tb and Y_2_O_3_: Eu exhibited ultra-fast luminescence response time (Fig. 1e and k). In particular, under 365 nm UV excitation, both Gd_2_O_2_S: Tb and Y_2_O_3_: Eu exhibited steep emission spectra that reached their peak intensity within 0.25 ms and 0.28 ms, respectively. Once the UV excitation was turned off, the peak emission intensity quickly decayed by 90% within 2.95 ms and 5.08 ms, respectively, with no detectable afterglow observed. Both scintillators showed no significant attenuation of the luminous intensity under prolonged X-ray irradiation (Fig. S3), suggesting their stable RL properties. Based on superior radioluminescence performances of Gd_2_O_2_S: Tb and Y_2_O_3_: Eu, we hypothesize that both scintillators can act as suitable transducers of X-ray to successfully activate the inhibitory opsins (ArchT and eNpHR 3.0) for wireless X-optogenetic neural inhibition.

### 3.2 Scintillators activate inhibitory opsins and induce hyperpolarization *in vitro*

**Fig. 2:**
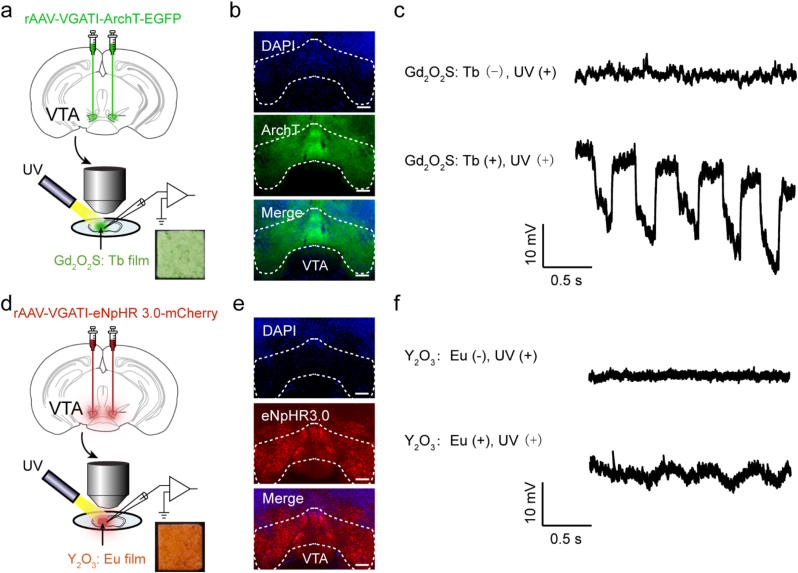
**Scintillators activate inhibitory opsins and induce neuronal hyperpolarization *in vitro*. (a)** Schematic of the patch-clamp experimental setup. A brain slice containing VTA neurons expressing the inhibitory ArchT is placed above a Gd_2_O_2_S: Tb-embedded film. UV light (365 nm) is used to excite the scintillator film, which emits green light to activate ArchT. **(b)** Representative confocal fluorescence images displaying the expression of ArchT-EGFP (green) in the VTA of a C57BL/6 mouse. Nuclei (DAPI, blue). Scale bars: 200 μm. **(c)** Representative voltage-clamp recording demonstrates robust membrane hyperpolarization (downward deflection) induced by PL of the Gd_2_O_2_S: Tb film under UV exposure. **(d)** A brain slice containing VTA neurons expressing eNpHR 3.0-mCherry is positioned over a Y_2_O_3_: Eu film. UV excitation of this film yields red light to activate eNpHR 3.0. **(e)** Representative confocal fluorescence images showing the expression of eNpHR 3.0-mCherry (red) in the VTA. Nuclei (DAPI, blue). Scale bars: 200 μm. **(f)** Representative voltage-clamp trace showing synchronous membrane hyperpolarization induced by PL from the Y_2_O_3_: Eu film under UV. (For interpretation of the references to colour in this figure legend, the reader is referred to the Web version of this article.)

We further performed patch-clamp recordings on acute brain slices to confirm whether both scintillators are able to activate the inhibitory opsins and induce neuronal hyperpolarization. Adeno-associated virus (serotype 2/9, AAV2/9) were stereotaxically injected into the ventral tegmental area (VTA) of wild-type C57BL/6 mice to drive the expression of the inhibitory opsins ArchT or eNpHR 3.0 (Fig. 2a and d) specifically on GABAergic neurons. Successful opsins expression in the VTA was confirmed by fluorescence microscopy four weeks post-injection (Fig. 2b and e). Due to the technical challenge of integrating an X-ray equipment directly with the patch-clamp setups, we employed a UV light source to optically excite the scintillators in these experiments, a validated proxy for simulating X-ray-induced radioluminescence.

Custom-made scintillator-embedded films were fabricated and placed beneath the brain slice during recording (Fig. 2a and d). Upon UV exposure, ArchT-expressing neurons exhibited pronounced membrane hyperpolarization (Fig. 2c), confirming effective activation of the ArchT by the emitted green light from Gd_2_O_2_S: Tb film. Furthermore, the membrane potential reliably tracked the ON-OFF cycles of the UV exposure, demonstrating precise temporal control over neuronal inhibition. Under the same UV exposure conditions, the Y_2_O_3_: Eu-embedded film similarly induced robust hyperpolarization in eNpHR 3.0-expressing neurons (Fig. 2f). Notably, the hyperpolarization amplitude was significantly greater in ArchT-expressing neurons when stimulated by Gd_2_O_2_S: Tb-embedded film, compared to that in eNpHR 3.0-expressing neurons activated by Y_2_O_3_: Eu films. This difference can be attributed to the wavelength-dependent photosensitivity of opsins. Although both scintillators emit light within the absorption bands of their respective opsins (Fig. 1d–h), the quantum efficiency of opsin activation varies with the specific emission peak of the scintillator.

Collectively, the *in vitro* patch-clamp results demonstrate that ArchT and eNpHR 3.0 can be efficiently and selectively activated by Gd_2_O_2_S: Tb and Y_2_O_3_: Eu scintillators, respectively, based on their visible light-emitting properties. Therefore, we propose that these Gd_2_O_2_S: Tb-ArchT and Y_2_O_3_: Eu-eNpHR 3.0 pairs are applicable to wireless deep-brain neural inhibition via X-ray-mediated scintillation *in vivo*.

### 3.3 X-optogenetic neural inhibition of VTA suppresses scratching behavior in freely moving mice

**Fig. 3:**
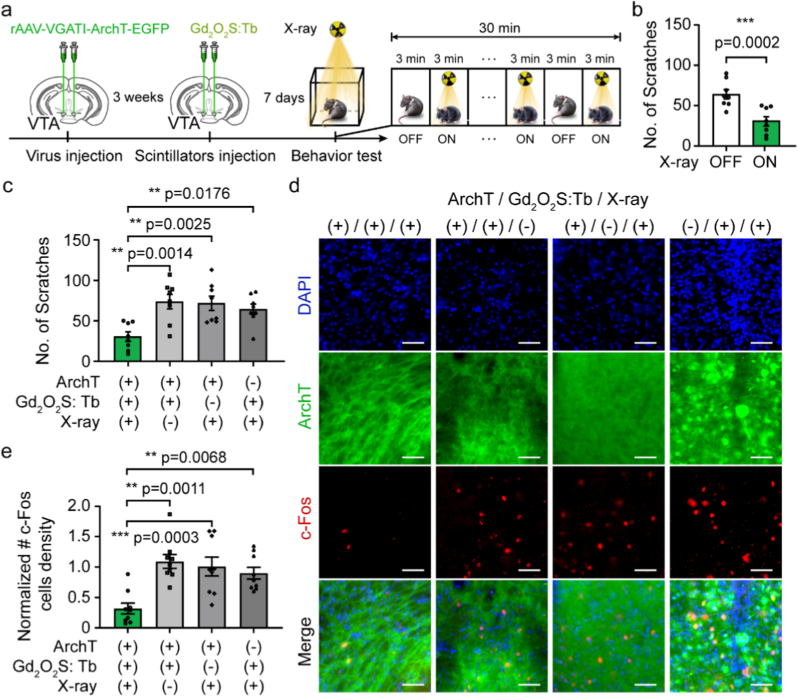
**Gd_2_O_2_S: Tb-mediated X-optogenetic neural inhibition of VTA suppresses scratching behavior in freely moving mice. (a)** Experimental timeline for viral and scintillator injections into the VTA and subsequent behavioral assessment during X-ray exposure. **(b)** Quantification of scratching bouts showing significant suppression in mice expressing ArchT in GABAergic neurons (ArchT (+)) and receiving Gd_2_O_2_S: Tb scintillator injection (Gd_2_O_2_S: Tb (+)) upon X-ray exposure (X-ray (+)) compared to baseline (n = 8 mice for each group, paired Student's *t*-test, ***p = 0.0002). (**c)** Changes in scratching behavior across different experimental conditions during the X-ray exposure period. (*n* = 8 mice for each group, two-way ANOVA with Tukey's multiple comparisons test). **(d)** Representative confocal images showing c-Fos expression (red) in tVTA neurons under different experimental conditions. GABAergic neurons are labeled with ArchT-EGFP (green). Scale bars: 500 μm. **(e)** Normalized density of c-Fos-positive cells in the VTA under different conditions (*n* = 9 brain slices for each group, *F*_*(3*_, _*32)*_ = 0.9425, *p* = 0.4316; one-way ANOVA). Error bars represent mean ± SEM. (For interpretation of the references to colour in this figure legend, the reader is referred to the Web version of this article.)

**Fig. 4:**
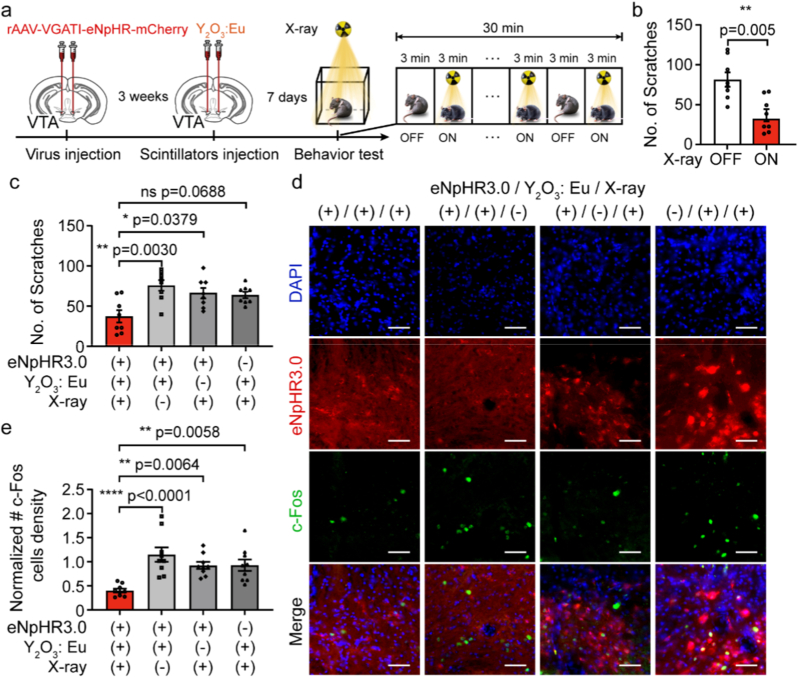
**Y_2_O_3_: Eu-mediated X-optogenetic neural inhibition of VTA suppresses scratching behavior in freely moving mice. (a)** Experimental timeline for stereotactic injection of AAV virus and Y_2_O_3_: Eu scintillators into the VTA of mice, followed byscratching behavior assessment during X-ray irradiation. **(b)** Reduction in scratching bouts observed in mice expressing eNpHR 3.0 (eNpHR 3.0 (+)) and receiving Y_2_O_3_: Eu (Y_2_O_3_: Eu (+)) upon X-ray exposure (X-ray (+)) (n = 8 mice for each group, paired Student's *t*-test, **p = 0.005). **(c)** Changes in scratching behavior during X-ray exposure under different conditions (*n* = 8 mice for each group, two-way ANOVA with Tukey's multiple comparisons tests). **(d)** Representative fluorescence images showing the expression of c-Fos (green) among eNpHR 3.0-mCherry-expresing GABAergic neurons (red) in the VTA of mice. Scale bars: 500 μm. **(e)** Normalized density of c-Fos-positive cells quantified from brain slices under different conditions (*n* = 9 brain slices for each group, *F*_*(3,32)*_ = 1.980, *p* = 0.1368; one-way ANOVA). Error bars represent mean ± SEM. (For interpretation of the references to colour in this figure legend, the reader is referred to the Web version of this article.)

To validate the efficacy of X-optogenetic neural inhibition technology *in vivo*, we performed the chloroquine-induced acute itch behavior investigations to confirm whether the technology can inhibit neural activity and suppress scratching bouts in awake, freely moving mice (Figs. 3a and 4a, and Fig. S5a). Given that optogenetic inhibition of GABAergic neurons in VTA has been established as an effective method to suppress scratching [62,63], we sought to replicate this effect using our wireless X-optogenetic platform.

To achieve robust and targeted opsin expression, we stereotactically injected adeno-associated virus (AAV2/9) carrying the genes for ArchT-EGFP or eNpHR 3.0-mCherry under the control of a GABAergic neurons-specific vesicular GABA transporter 1synapsin-1 promoter (VGAT1) into the VTA of wild-type C57BL/6 mice.

Three weeks post-AAV injection, the same mice received a stereotactic injection of Gd_2_O_2_S: Tb into the VTA. The AAV2/9-EGFP and saline were injected at the same position in control groups. All acute itch mice were adapted to the experimental cage for 30 min to minimize animal's anxiety. Following 30-min acclimation period, mice were subjected to a specific X-ray exposure protocol (150 keV, 0.5 mA, 3-min pulses every 6 min, five cycle; dose rate: 0.186 Gy/min) while their scratching behavior in the observation arena was recorded.

During X-ray exposure, mice in experimental group (ArchT (+), Gd_2_O_2_S: Tb (+), X-ray (+)) exhibited a 52.24% reduction in total scratching bouts (from 64.13 ± 5.84 to 30.63 ± 5.82), compared to their baseline in the period before X-ray stimulation (Fig. 3b). In contrast, no significant change in scratching behavior was found in any of the control groups, including those lacking one of the three critical components (ArchT (+), Gd_2_O_2_S: Tb (+), X-ray (−): from 76.13 ± 8.54 to 73.75 ± 8.96; ArchT (+), Gd_2_O_2_S: Tb (−), X-ray (+): from 70.13 ± 8.73 to 71.63 ± 8.78; ArchT (−), Gd_2_O_2_S: Tb (+), X-ray (+): from 61.13 ± 8.23 to 64.25 ± 6.49), as shown in Fig. 3c and Fig. S6a.

To correlate this scratching behavior suppression with neuronal activity, we conducted immunofluorescence analysis for c-Fos, an immediate early gene marker of neuronal activity. Consistent with the behavioral data, the experimental group (ArchT (+), Gd_2_O_2_S: Tb (+), X-ray (+)) showed a significantly lower normalized density of c-Fos^+^ cells (0.32 ± 0.09) in the VTA compared to all control groups (ArchT (+), Gd_2_O_2_S: Tb (+), X-ray (−): 1.09 ± 0.11; ArchT (+), Gd_2_O_2_S: Tb (−), X-ray (+): 1.01 ± 0.15; ArchT (−), Gd_2_O_2_S: Tb (+), X-ray (+): 0.90 ± 0.10; normalized) (Fig. 3d and e, Movie S1). Notably, a significant suppression of scratching bouts was observed in the experimental group even during the first X-ray stimulation period, while no significant change was observed in other groups, further confirming the rapid onset of the X-optogenetic inhibitory effect (Fig. S7a–S7d). Collectively, these results demonstrate that the Gd_2_O_2_S: Tb-ArchT pair can achieve effective GABAergic neuronal inhibition in the VTA, which directly translates into a significant suppression of itch-induced scratching behavior in freely moving mice.

To verify the broad applicability our wireless X-optogenetics platform, a second series of experiments was conducted utilizing the inhibitory chloride pump eNpHR 3.0 paired with the red-emitting Y_2_O_3_: Eu scintillator. An AAV2/9 virus expressing eNpHR 3.0-mCherry under the GABAergic neuron-specificVGAT1 promoter was stereotactically into the VTA of C57BL/6 mice (Fig. 4a). Three weeks later, Y_2_O_3_: Eu scintillators were injected into the same brain region. Control group received AAV2/9-mCherry and saline at the same sit. Behavioral assessments during X-ray exposure were performed under an identical protocol as described for the Gd_2_O_2_S:Tb-ArchT experiments (150 keV, 0.5 mA; 3-min on/6-min off cycles, 5 times).

Only mice in experimental group during X-ray stimulation (eNpHR 3.0 (+), Y_2_O_3_: Eu (+), X-ray (+)) exhibited a robust and specific reduction in itch-induced scratching. This group showed a 52.53% decrease in scratching bout (from 86.63 ± 7.67 to 41.13 ± 8.69; mean ± SEM) during X-ray exposure (Fig. 4b). In contrast, no statistically significant behavioral suppression was found in the control groups (eNpHR 3.0 (+), Y_2_O_3_: Eu (+), X-ray (−): from 80.63 ± 9.44 to 76.13 ± 6.66; eNpHR 3.0 (+), Y_2_O_3_: Eu (−), X-ray (+): from 76.75 ± 7.57 to 66.5 ± 6.50; eNpHR 3.0 (−), Y_2_O_3_: Eu (+), X-ray (+): 72.88 ± 7.67 to 63.88 ± 3.96; Fig. 4c, Fig. S6b, Movie S2). Consistent with the behavioral outcomes, neuronal activity in the VTA, measured via c-Fos immunofluorescence analysis, was specifically inhibited in the experimental group (eNpHR 3.0 (+), Y_2_O_3_: Eu (+), X-ray (+)). The normalized density of c-Fos-positive cells was significantly lower (0.40 ± 0.04) compared to all other groups (eNpHR 3.0 (+), Y_2_O_3_: Eu (+), X-ray (−): 1.15 ± 0.15; eNpHR 3.0 (+), Y_2_O_3_: Eu (−), X-ray (+): 0.92 ± 0.07; eNpHR 3.0 (−), Y_2_O_3_: Eu (+), X-ray (+): 0.93 ± 0.12), as presented in Fig. 4d and e. Further analysis of the initial stimulation period revealed that while a statistically significant reduction was not yet achieved during the first X-ray pulse across all groups, a strong trend toward suppression was present exclusively in the experimental group (*p* = 0.1164, paired *t*-test, Fig. S7e–h). It suggests a rapid inhibitory effect, which becomes pronounced and significant over the full course of stimulation. Collectively, the Y_2_O_3_: Eu-eNpHR 3.0 pairs can sufficiently inhibit the GABAergic neural activities in the VTA and suppress the scratching behavior in freely moving mice.

We also analyzed the time course of changes in mouse scratching bouts. As shown in Fig. S8, for mice injected with ArchT and Gd_2_O_2_S: Tb, starting from the ON 2 period (corresponding to 9–12 min), the cumulative scratching bouts of all contiguous periods showed statistical significance compared to each other. Ift indicates that the difference in scratching bouts between radiation ON and OFF periods became increasingly significant as time progressed. From 9 to 12 min onward, the total scratching bouts of the mice during the radiation-on state was significantly reduced. For mice injected with eNpHR 3.0 and Y_2_O_3_: Eu, starting from the ON 2 period (9–12 min), each time the radiation was turned off, the scratching frequency in the subsequent period significantly increased, indicating effective suppression of scratching behavior during the radiation-on period. However, the data for each ON period did not show a significant difference compared to the preceding OFF period. This suggests that under our experimental conditions, the combination of eNpHR 3.0 and Y_2_O_3_: Eu exhibited slightly weaker neural inhibition compared to the “ArchT and Gd_2_O_2_S: Tb” treatment.

We also performed experiments and evaluated the background luminescence that could interfere with X-optogenetic neural inhibition. Under X-ray irradiation (150 kV, 0.5 mA) used in X-optogenetics, we tested the luminescence intensity of different samples (intact brain tissues and coronal brain sections of mice) by a high-sensitivity, low-noise charge-coupled device (CCD) optical camera in the X-ray luminescence computed tomography (XLCT) measurement system. The results demonstrated that no significant fluorescence signal can be found on brain tissue (Fig. S9 and Fig. S10) or brain sections without scintillator injection (Fig. S11f and Fig. S11l). Notably, clear radioluminescence signal was observed only in images on brain sections of mice, including the (ArchT (+), Gd_2_O_2_S: Tb (+), X-ray (+)) and (eNpHR 3.0 (+), Y_2_O_3_: Eu (+), X-ray (+)) groups (Fig. S11c and Fig. S11i). These results confirmed that background luminescence under X-ray irradiation did not interfere with opsin activation or neural inhibition.

### 3.4 Biosafety assessment of scintillator-mediated X-optogenetic technology

We measured the cytotoxicity of Gd_2_O_2_S: Tb and Y_2_O_3_: Eu scintillators *in vitro* using HEK293 cells, as commonly used in related fields such as magnetogenetics [38] and ultrasound-optogenetics [39]. Cell viability assays demonstrated that at the scintillator concentrations up to 50 μg/mL, neither Gd_2_O_2_S: Tb nor Y_2_O_3_: Eu induce significant cytotoxicity. The viability of cells treated with scintillators remained comparable to that of the control group without scintillator (Fig. S4a and S4b). These results indicate that both scintillators possess favorable biocompatibility, supporting their suitability for X-optogenetic applications *in vitro* and *in vivo*.

We further evaluated the *in vivo* safety of our scintillator-mediated X-optogenetics platform at a total dose of 2.78 Gy. Firstly, this dose is significantly lower than the established threshold reported to induce acute neuronal dysfunction, change locomotor behavior, or compromise blood-brain barrier integrity in rodent mice, and is substantial below standard clinical radiotherapy doses [46,[64], [65], [66]]. Consistent with this, our behavioral data confirm that this irradiation protocol did not elicit significant changes in spontaneous or itch-induced scratching in any of the control groups. Furthermore, all animals subjected to a single irradiation session exhibited normal survival and body weight trajectories over a follow-up period of at least 8 weeks, supporting the feasibility of this X-optogenetics technology for long-term wireless neurostimulation.

**Fig. 5:**
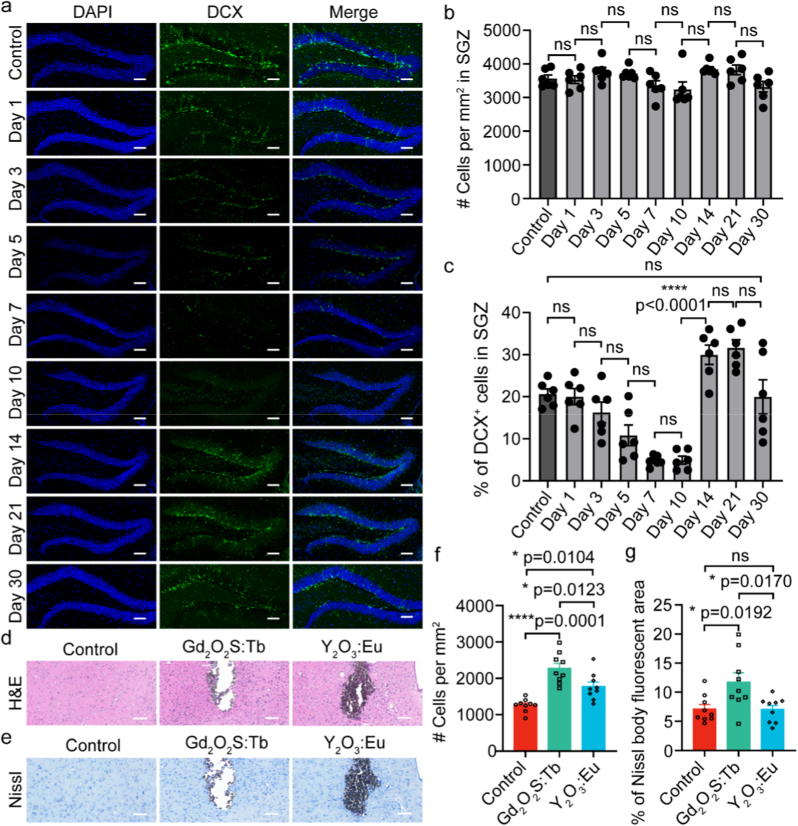
**Biosafety assessment of scintillator-mediated X-optogenetics. (a)** Representative fluorescence images of DAPI (blue) and doublecortin (DCX, green) at the hippocampal dentate gyrus. Images were acquired from mice without radiation exposure (Control group) and on Day 1, Day 3, Day 5, Day 7, Day 10, Day 14, Day 21, and Day 30 following a single 2.78 Gy radiation session. Scale bars: 100 μm. **(b)** Quantification of cell density within the subgranular zone (SGZ, *n* = 6 brain slices for each group, *F*_(8, 45)_ = 0.4810, *p* = 0.8632; one-way ANOVA). **(c)** Quantification of DCX-positive (DCX^+^) cells percentage in SGZ under different conditions (*n* = 6 brain slices for each group, *F*_(8, 45)_ = 2.964, *p* = 0.0094; one-way ANOVA). **(d)** Representative H&E staining images and **(e)** Nissl staining images from the corresponding brain regions of the control group (left, no scintillator injection), Gd_2_O_2_S: Tb-injected group (middle), and Y_2_O_3_: Eu-injected group (right). Scale bars: 100 μm. **(f)** Quantification of neuronal density around the scintillator injection site based on Nissl staining (*n* = 9 regions for each group, *F*_(2, 24)_ = 2.201, *p* = 0.1325; one-way ANOVA). **(g)** Quantification of the percentage of Nissl body fluorescent area (*n* = 9 regions for each group, *F*_(2, 24)_ = 1.800, *p* = 0.1869; one-way ANOVA). (For interpretation of the references to colour in this figure legend, the reader is referred to the Web version of this article.)

We also assessed potential impact on neurogenesis, a process highly sensitive to ionizing radiation, through immunohistochemical staining of doublecortin (DCX) (Fig. 5a), a marker of immature neuronal cells, in the hippocampal dentate gyrus [46]. Because the whole-body exposure to X-rays is known to damage radiosensitive cells. No significant difference in the total number of cells was found within the dentate gyrus between irradiated mice and age-matched, non-irradiated mice in control groups (Fig. 5b). However, quantitative results revealed an obvious decrease in the proportion of DCX-positive cells on day 7 post-irradiation, indicating a partial and acute suppression of hippocampal neurogenesis. Importantly, this proportion of immature neurons (DCX-positive neurons) had recovered to baseline levels on day 14 (Fig. 5c), demonstrating the resilience and complete functional restoration of the neurogenic niche within one month. Collectively, these findings indicate that the X-ray dose (2.78 Gy) utilized in our protocol induces only minimal and fully reversible functional impairment, without compromising the endogenous repair capacity of the brain.

Hippocampal neurogenesis primarily involves the generation, maturation, and integration of new neurons over weeks to months [67]. Briefly, hippocampal neurogenesis produces dentate gyrus (DG) granule cells (GCs) from quiescent radial glia-like stem cells (Type I, SOX2/GFAP-positive). Activated Type I cells generate intermediate progenitors that mature into neuroblasts, then functional GCs (morphologically and electrophysiologically mature), and interate into circuits over weeks to months. Temporary suppression of hippocampal neurogenesis may temporarily impair memory formation and emotional regulation, but does not lead to permanent functional loss [68].‌ Because hippocampal function relies on the activity of existing neural networks, and function is typically reversible after short-term suppression [69]. In our X-optogenetics, the proportion of immature neurons recovered to baseline levels within 30 days, indicating the resilience and functional restoration of the hippocampal neurogenesis. Even so, testing the hippocampal functional implications represents an important direction for future studies.

The local tissue response to the injected scintillators was evaluated using hematoxylin and eosin (H&E) and Nissl staining. As shown in H&E-stained images (Fig. 5d), both Gd_2_O_2_S:Tb and Y_2_O_3_: Eu scintillators were well dispersed along the periphery of the injection track, without notable aggregation. No obvious neuronal morphological changes or cell death were observed in cells adjacent to either scintillator. A mild gliotic response was found around both scintillator types, compared with the control group. Sporadic localized calcifications (up to 55 × 15 μm) were identified surrounding the Gd_2_O_2_S: Tb particles, which were absent in control group. In Nissl staining, while an increase in Nissl body number was noted in proximity to the scintillators, no significant signs of acute neuronal damage (e.g., chromatolysis) were observed within a 50 μm radius (Fig. 5e). Quantitative analysis of neuronal density in the peri-injection area revealed that regions injected with Gd_2_O_2_S: Tb scintillators exhibited the highest density (2269.83 ± 139.19 neurons per mm^2^), followed by Y_2_O_3_: Eu-injected areas (1770.318 ± 124.07 neurons per mm^2^), with the control region showing the lowest density (1259.56 ± 59.49 neurons per mm^2^), as presented in Fig. 5f. Similarly, the percentage of Nissl-positive area was significantly greater around the Gd_2_O_2_S: Tb sites (11.72 ± 1.61 %) compared to both the control group (7.10 ± 0.79 %) and the Y_2_O_3_: Eu-injected group (7.01 ± 0.72 %). No significant difference was observed between the Y_2_O_3_: Eu group and the control group (Fig. 5g). These findings suggest that the Y_2_O_3_: Eu exhibits superior biocompatibility compared to the Gd_2_O_2_S: Tb. Nevertheless, it is important to note that both scintillators demonstrate good biocompatibility and suitability for *in vivo* X-optogenetic applications.

## 4 Conclusion and future perspectives

We demonstrated an effective wireless deep-brain neural inhibition technology in awake, freely moving mice using two novel scintillator-opsins pairs (Gd_2_O_2_S: Tb-ArchT and Y_2_O_3_: Eu-eNpHR 3.0) under low-dose X-ray exposure. Our electrophysiological recordings, c-Fos-induction experiments, and behavioral experiments collectively verified that the scintillator-opsins pairs could quickly inhibit the activity of GABAergic neurons in VTA and suppress itch-evoked scratching behavior in mice.

Recent optogenetic neuronal inhibition has been proven to be feasible [70,71], yet few reports focus on wireless optogenetic neural inhibition. Our wireless X-optogenetic neural inhibition technology based is suitable for remote inhibition of neural activity in awake moving animals. Notably, for managing numerous neurological disorders, wireless neural inhibition in deep brain is a promising approach [72], which employs external energy sources and eliminates implanted rigid devices, thereby minimizing tissue damage. Compared to NIR light, ultrasound, and magnetic fields, X-ray provides exceptional tissue penetration (about 30 cm), high spatial resolution (about 0.1 mm), and minimal thermal effects [42], making it suitable and preferred for deep-brain stimulations. Moreover, this technology holds promise to inhibit neural activities for therapying neurological disorders, such as epilepsy [[73], [74], [75], [76], [77]].

High-dose X-rays can directly damage DNA molecules or generate free radicals that cause indirect damage. It is because high X-ray energy can knock electrons out of atoms, creating charged particles (ions) that disrupt cellular structures. We have conducted some experiments to test different X-ray doses and find a reasonable experimental parameter. As shown in Fig. S12, when the X-ray doses was set to 0.57 Gy and 1.72 Gy, there was no significant change in the scratching behavior of the mice. However, when the doses were set to 2.78 Gy and 4.02 Gy, the scratching behavior significantly decreased during radiation. Since a higher dose raises biosafety concerns, we discarded the 4.02 Gy parameter. Ultimately, we determined that a dose of 2.78 Gy is sufficient to induce significant behavioral changes. The total dose (2.78 Gy) used in our experiments is significantly lower than that used in the previously reported X-optogenetics and clinical radiotherapy [46]. Importantly, at 2.78 Gy, no adverse effects on mouse viability were observed. And a significant behavioral effect was evident even at a ‌cumulative dose of only 0.56 Gy‌ (one-fifth of the total; Fig. S7a). Reducing the dose of X-ray irradiation is key to enhancing safety and enabling long-term, repeated X-optogenetic neurostimulation. And Low dose is also the precondition of clinical translation. Precisely confining the X-ray beam using collimators on the target brain region and real-time tracking systems would eliminate whole-body exposure. Simpler methods, such as shielding non-target areas with lead plates, optimizing irradiation voltage and distance, could also protect nontargeted regions from X-ray irradiation.

Newly reported inhibitory opsins that can be activated by pulsed light stimulation are capable of inducing sustained neuronal hyperpolarization [73]. Inhibitory opsins (e.g., ArchT, eNpHR 3.0) used in this study typically require sustained illumination, leading to higher dose requirements compared to excitatory opsin activated by pulsed light illumination. Employing X-ray beam choppers that can change irradiation frequency could drastically minimize the irradiation dose required for activating opsins.

Further dose reduction is achievable through developing novel scintillators with excellent RL performances. The light yield of scintillators is mainly influenced by their intrinsic nature, such as particle size, composition, and morphology. Among them, the particle size of scintillators is a key factor. It is because nano-sized scintillators exhibit obviously improved biocompatibility, targeting ability, and permeability within biological tissues, but slightly diminished luminescent efficiency [45,78,79]. The design of high-light-yield nano-scintillators, such as rare-earth-doped nanoparticles, may be another viable option to enable precise and effective X-optogenetic neuromodulation [80,81].

Current X-optogenetics technology has only been demonstrated in mice. The most outstanding advantage of X-optogenetics lies in the unparalleled penetration depth and long-distance irradiation ability of X-rays compared to other wireless optogenetic technologies. In the future, this technology may serve as a powerful wireless deep-brain stimulation tool for neuroscience investigations and neurological disorder therapies in large living models.

## CRediT authorship contribution statement

**Bin Lan:** Conceptualization, Data curation, Investigation, Methodology, Visualization, Writing – original draft, Writing – review & editing. **Haiying Liu:** Data curation, Investigation, Methodology. **Jinwei Xu:** Investigation, Methodology. **Yifan Zhang:** Writing – review & editing. **Dongyan Li:** Investigation. **Peng Gao:** Writing – review & editing. **Wenli Zhang:** Writing – review & editing. **Wenting Wang:** Supervision, Visualization, Writing – review & editing. **Galong Li:** Funding acquisition, Writing – review & editing. **Hongbing Lu:** Conceptualization, Funding acquisition, Project administration, Supervision, Writing – review & editing.

## Declaration of competing interest

The authors declare that they have no known competing financial interests or personal relationships that could have appeared to influence the work reported in this paper.

## Data Availability

Data will be made available on request.
