# Supplementary material for: X-optogenetic inhibition of VTA GABA neurons for wireless deep-brain stimulation and suppression of scratching behavior in freely moving mice
Source: Mater Today Bio. 2026 Apr 17;38:103137. doi: 10.1016/j.mtbio.2026.103137 (PMC13101772; doi:10.1016/j.mtbio.2026.103137)
Supplement: Multimedia component 1 [file mmc1.docx]

Supporting information for

**X-optogenetic inhibition of VTA GABA neurons for wireless deep-brain stimulation and suppression of scratching behavior in freely moving mice**

Bin Lan^1,#^, Haiying Liu^2,#^, Jinwei Xu^2,#^, Yifan Zhang^3^, Dongyan Li^1^, Peng Gao^1^, Wenli Zhang^1^, Wenting Wang^2, *^, Galong Li^1, *^, Hongbing Lu^1, *^

^1^ School of Biomedical Engineering, Air Force Medical University, Xi’an 710032, China

^2^ Department of Neurobiology, School of Basic Medicine, Air Force Medical University, Xi'an, China

^3^ Institute for Chinese Medicine Frontier Interdisciplinary Science and Technology, Shaanxi University of Chinese Medicine, Xixian New Area, Shaanxi Province, 712046, China

^*^ Correspondence: Wenting Wang, wwt0657@fmmu.edu.cn; Galong Li, ligalong@fmmu.edu.cn; Hongbing Lu, luhb@fmmu.edu.cn.

^#^ These authors contributed equally to this article.


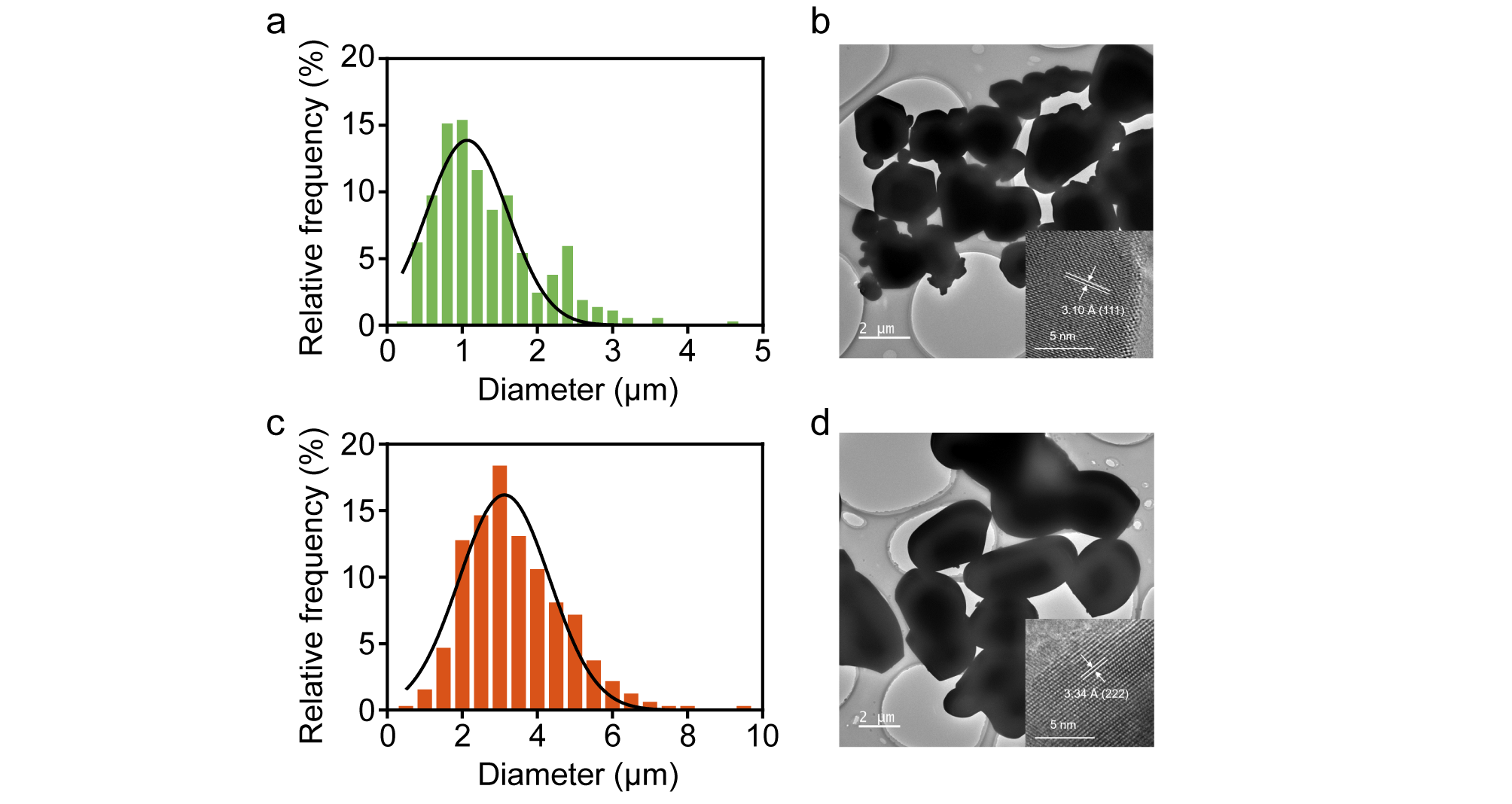


**Figure S1.** **(a)** Particle size distribution and **(b)** TEM of Gd_2_O_2_S: Tb scintillators. **(c)** Particle size distribution and **(d)** TEM of Y_2_O_3_: Eu scintillators. Insets of TEM depict the corresponding HR-TEM.


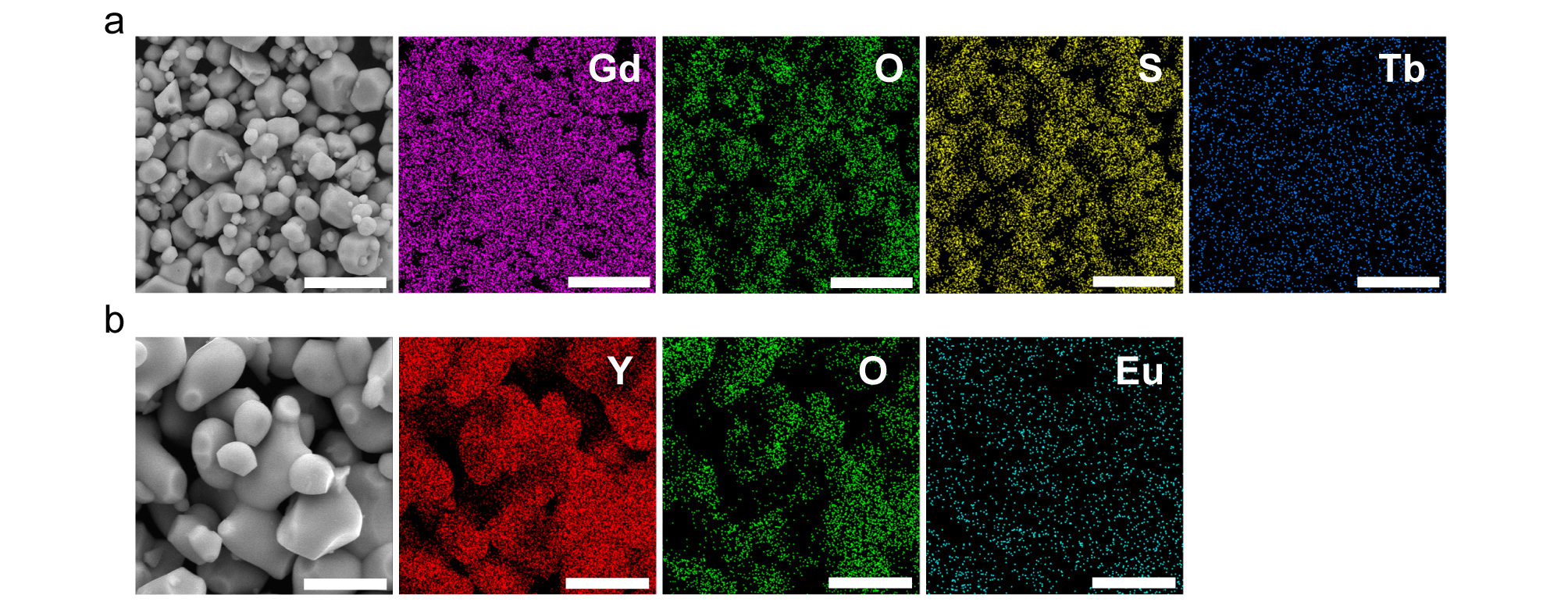


**Figure S2. Energy-dispersive X-ray spectroscopy (EDS) of scintillators. (a)** EDS elemental mapping of Gd₂O₂S: Tb, showing the distributions of O (green), S (yellow), Gd (purple), and Tb (blue). **(b)** EDS elemental mapping of Y₂O_3_: Eu, showing the distributions of O (green), Y (red), and Eu (blue).

**Table 1.** Elemental composition of Gd₂O₂S: Tb scintillator.

| **Element** | **Weight (%)** | **Atomic (%)** |
| --- | --- | --- |
| O | 7.56 | 38.54 |
| S | 6.69 | 17.01 |
| Gd | 84.52 | 43.82 |
| Tb | 1.22 | 0.63 |
| Total | 100.00 | 100 |

**Table 2.** Elemental composition of Y₂O_3_: Eu scintillator.

| **Element** | **Weight (%)** | **Atomic (%)** |
| --- | --- | --- |
| O | 19.45 | 58.17 |
| Y | 73.67 | 39.66 |
| Eu | 6.88 | 2.17 |
| Total | 100.00 | 100 |


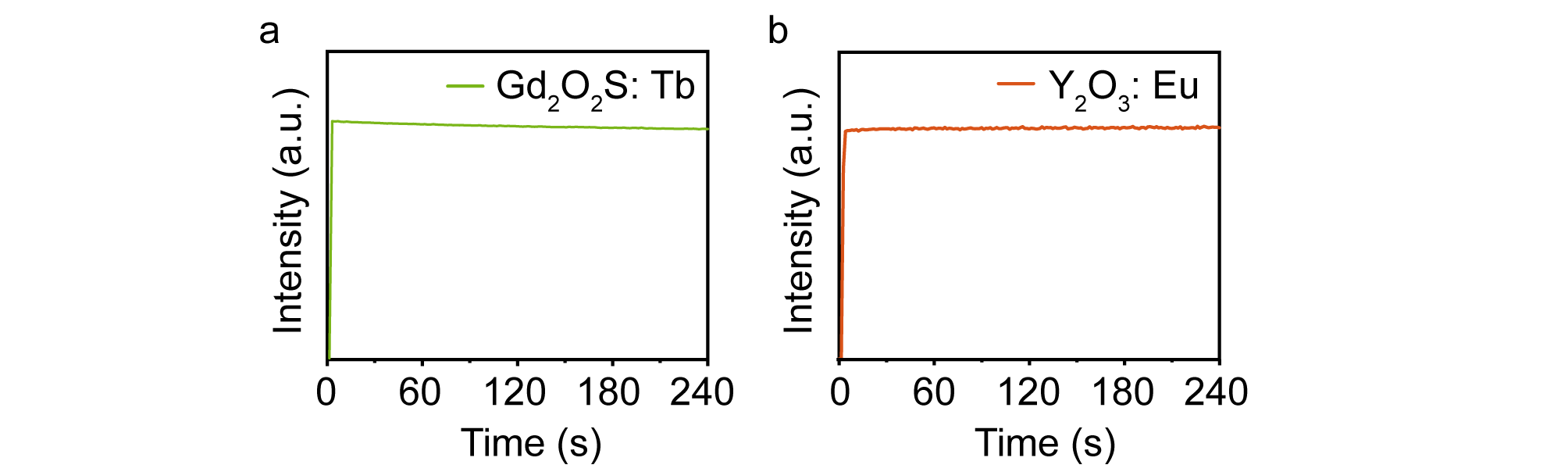


**Figure S3.** Stable luminescence of **(a)** Gd₂O₂S: Tb and **(b)** Y₂O_3_: Eu under X-ray excitation.


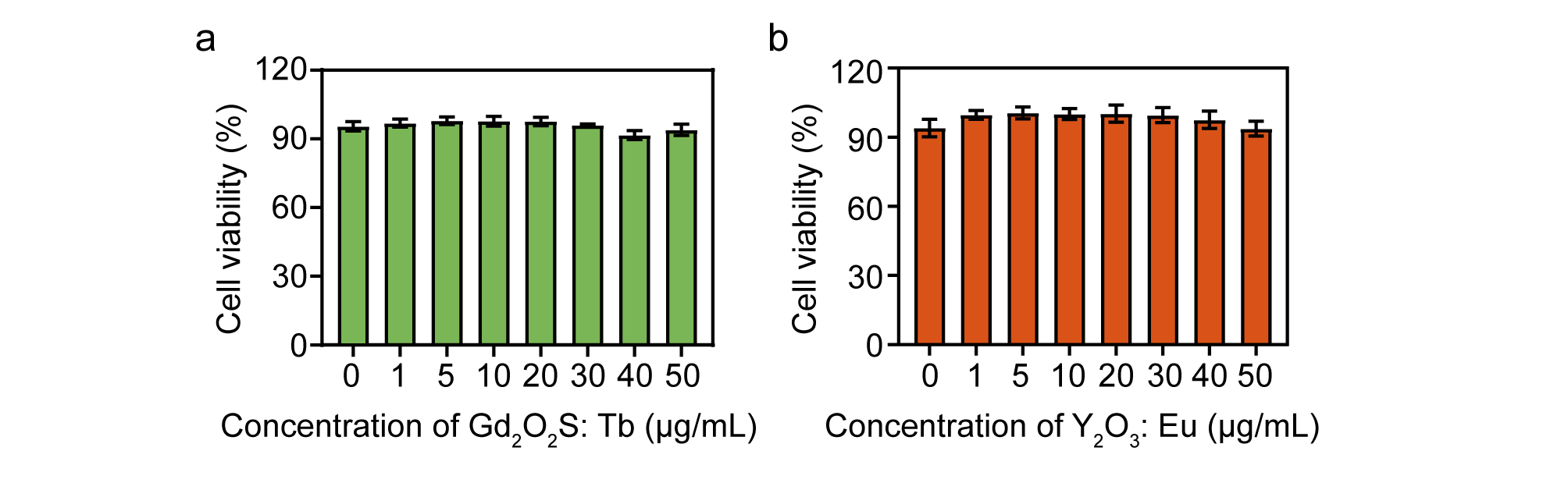


**Figure S4. Cell viability of scintillators.** The cell viability of HEK293 cells cultured with **(a)** Gd_2_O_2_S: Tb or **(b)** Y_2_O_3_: Eu at different concentrations from 0 to 50 μg mL^-1^ within the cells' growth media.


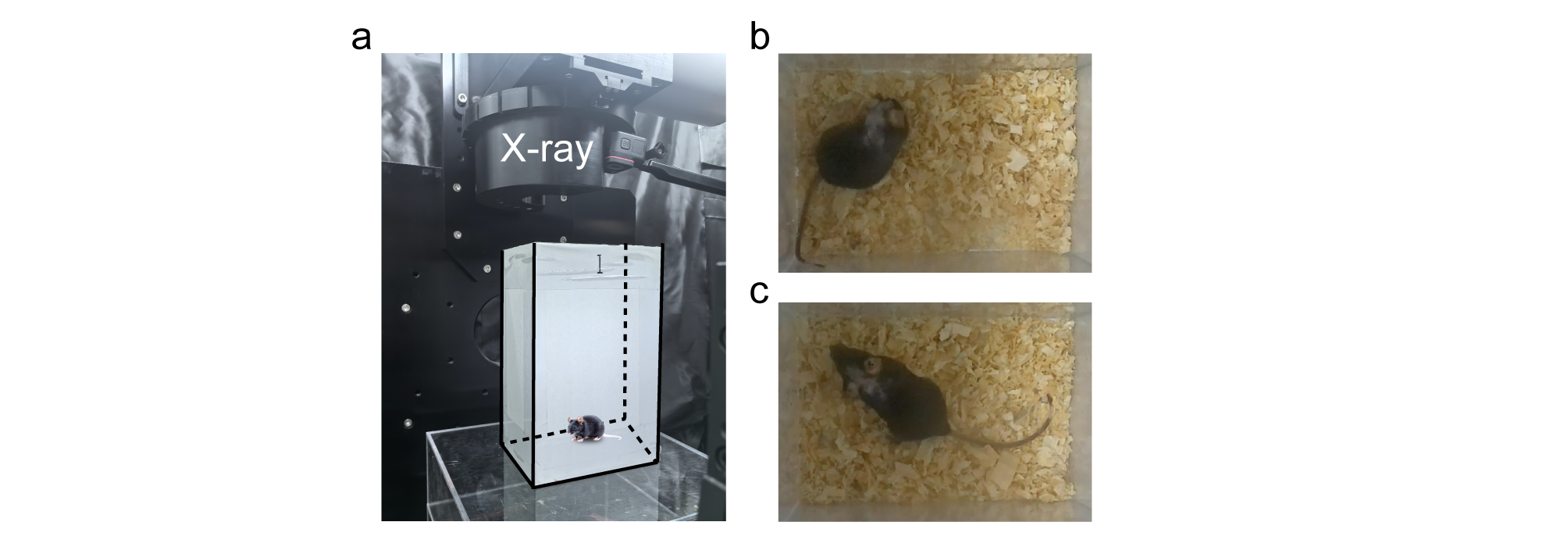


**Figure S5. Itch-induced scratching behavior experiments. (a)** A photograph of the X-ray setup used for in vivo experiments. **(b)** Scratching behavior of mice in the absence of X-ray stimulation. **(c)** Suppressed scratching behavior of mice during X-ray stimulation.


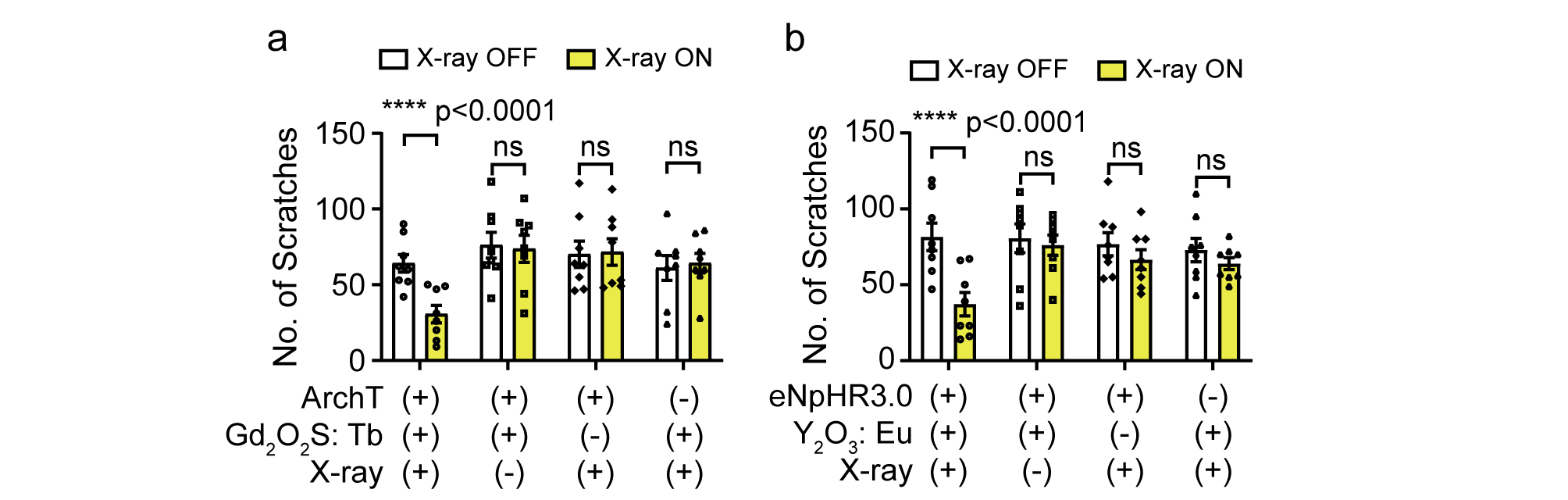


**Figure S6. Scratching behavior changes in mice. (a)** Mice expressing ArchT in the VTA neurons (ArchT(+)) and injected with Gd₂O₂S: Tb (Gd₂O₂S: Tb(+)) exhibited suppressed scratching behavior in response to the X-ray stimulus (*n* = 8 mice for each group, two-way ANOVA with Tukey’s multiple comparisons test). **(b)** Mice expressing eNpHR 3.0 in the VTA neurons (eNpHR 3.0 (+)) and injected with Y₂O_3_: Eu (Y₂O_3_: Eu(+)) exhibited suppressed scratching behavior in response to the X-ray stimulus (*n* = 8 mice for each group, two-way ANOVA with Tukey’s multiple comparisons test).


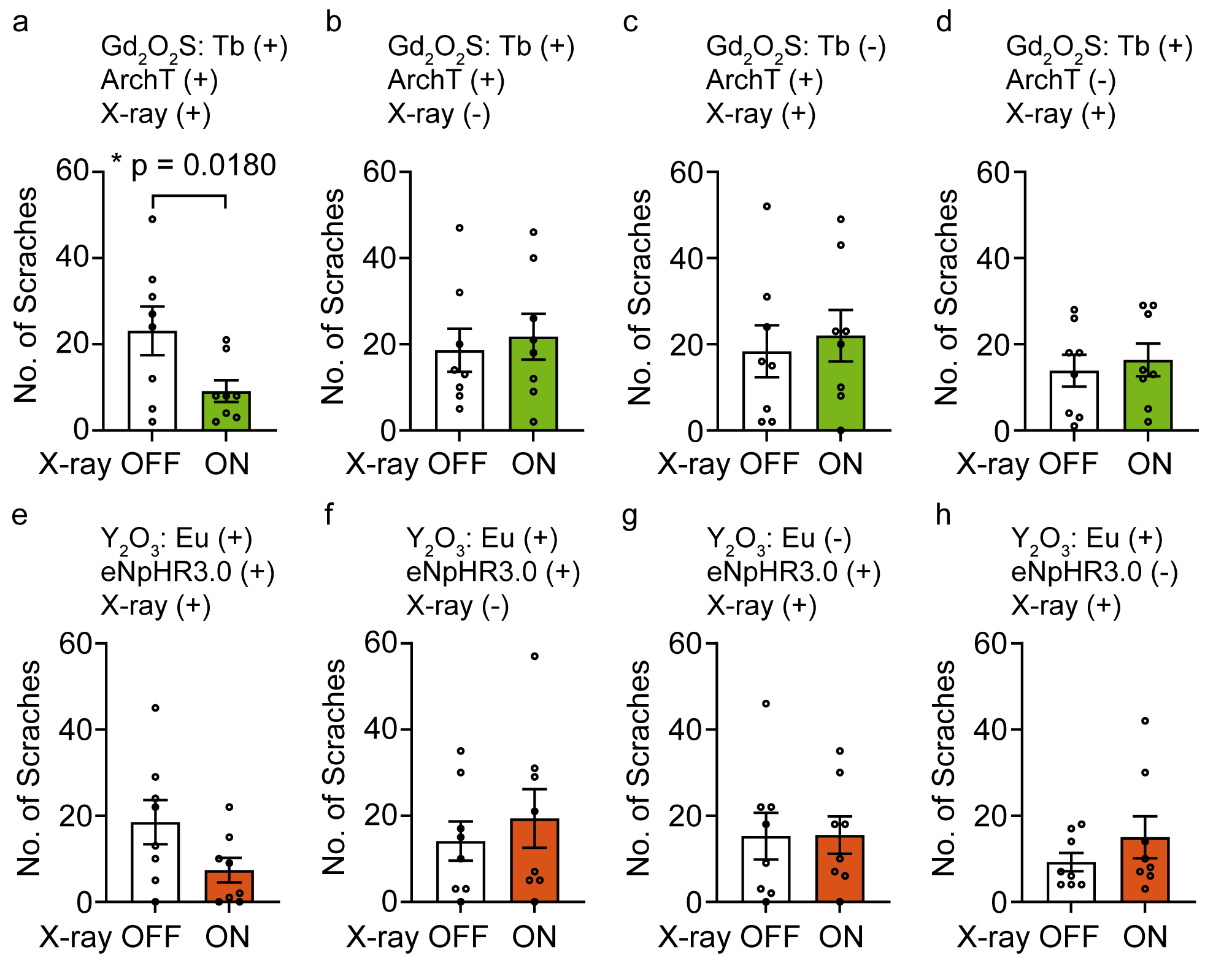


**Figure S7. Scratching behavior changes in mice. (a-d)** Comparison of scratching bouts in mice during the first 6 minutes of the ArchT-Gd₂O₂S: Tb experiment. The OFF period (no X-ray stimulation) corresponds to minutes 1-3, while the ON period (with X-ray stimulation) corresponds to minutes 4-6 of the experimental session (n = 8 mice for each group, paired t-test). **(e-h)** Comparison of scratching bouts in mice during the first 6 minutes of the eNpHR 3.0-Y₂O_3_: Eu experiment. The OFF period (no X-ray stimulation) corresponds to minutes 1-3, while the ON period (with X-ray stimulation) corresponds to minutes 4-6 of the experimental session (n = 8 mice for each group, paired t-test).


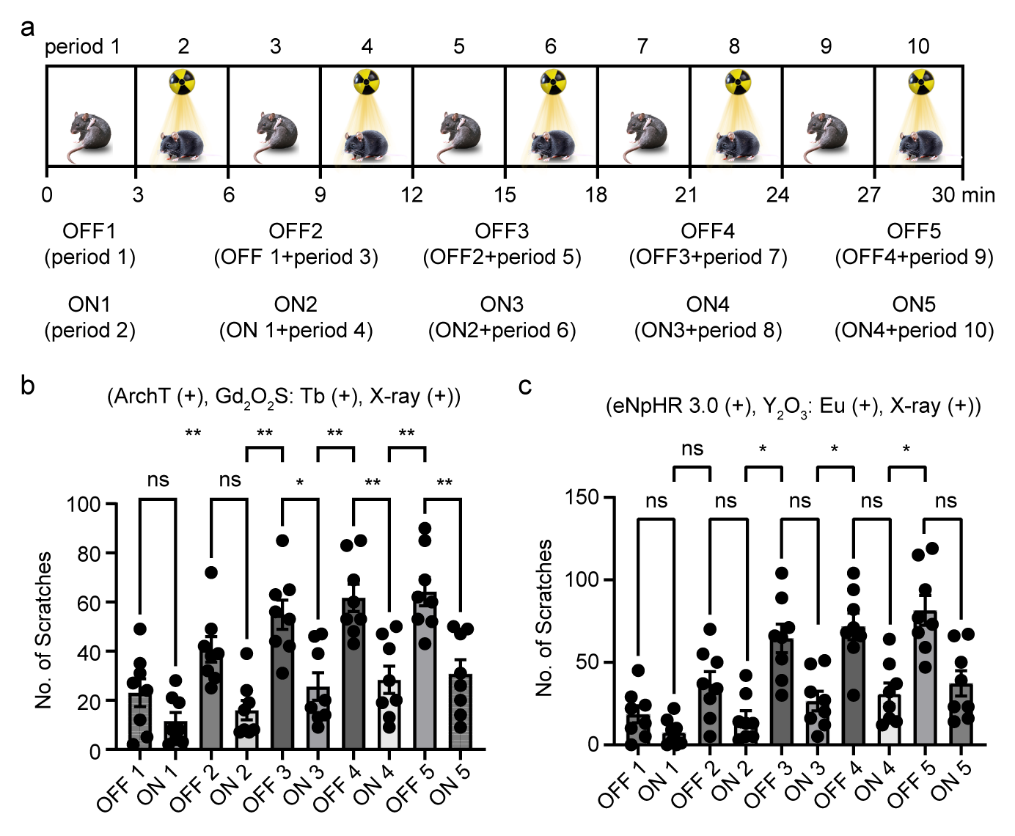


**Figure S8.** **Analysis of the time course of behavioral suppression.** **(a)** Procedure of a complete experiment over 30 min. **(b)** Comparison of cumulatively summed scratching bouts of mice (ArchT (+), Gd_2_O_2_S: Tb(+)) over 30 min. This group of mice was injected with. **(c)** Comparison of cumulatively summed scratching bouts of mice (eNpHR 3.0(+), Y_2_O_3_: Eu(+)) over 30 min. OFF: without X-ray irradiation; ON: with X-ray irradiation.


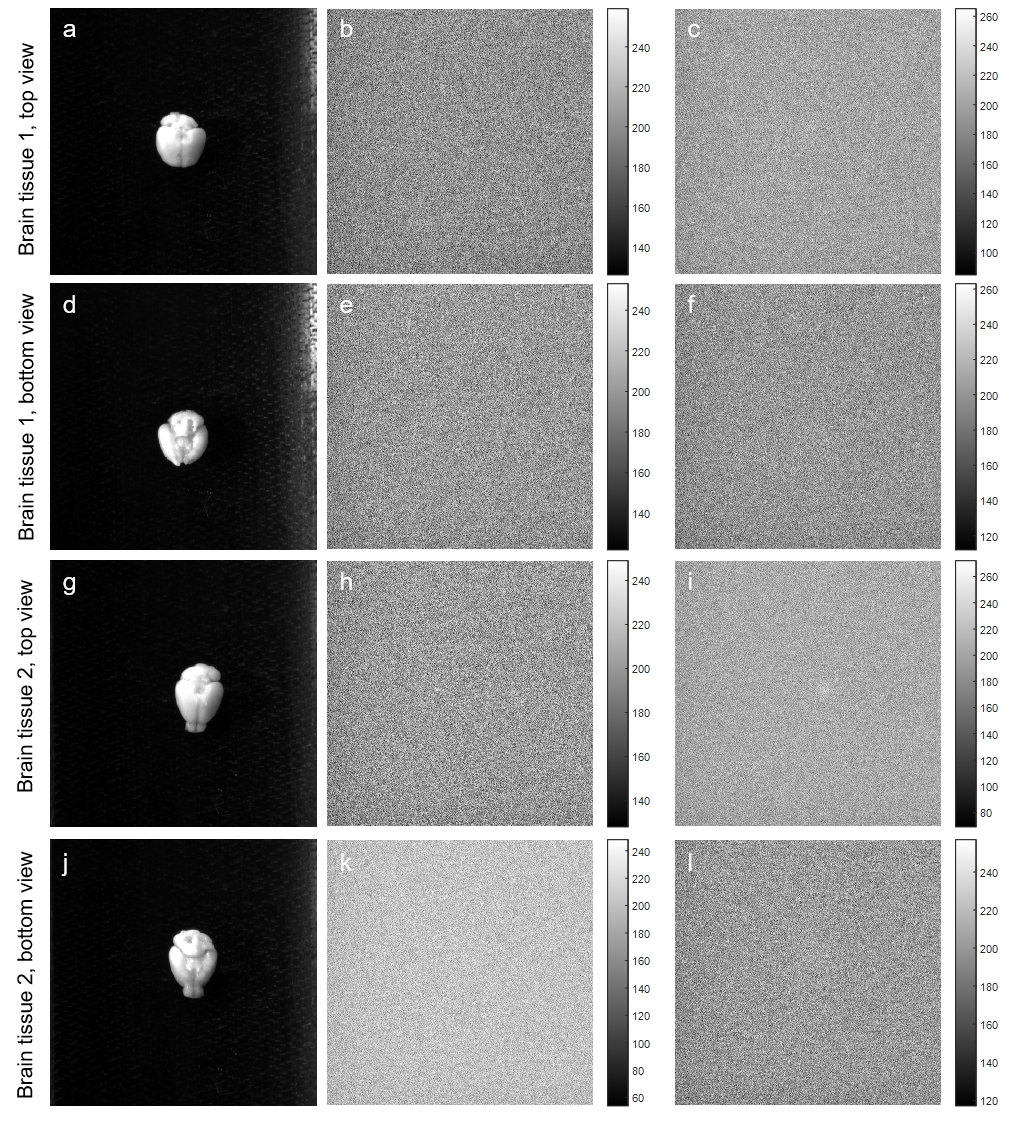


**Figure S9. Fluorescence test of intact brain tissues.** Positioning photograph of the **(a)** top view and **(d)** bottom view of brain tissue 1 (ArchT(+), Gd_2_O_2_S: Tb(+)) under white light. Photograph of the **(b)** top view and **(e)** bottom view of brain tissue 1 (fluorescence background) in a darkroom. Photograph of the **(c)** top view and **(f)** bottom view of brain tissue 1 (radioluminescence) under X-ray irradiation. Photographs of brain tissue 2 (ArchT(+), Gd_2_O_2_S: Tb(-)) were displayed in **(g-l)**, with the same layout as **(a-f)**.


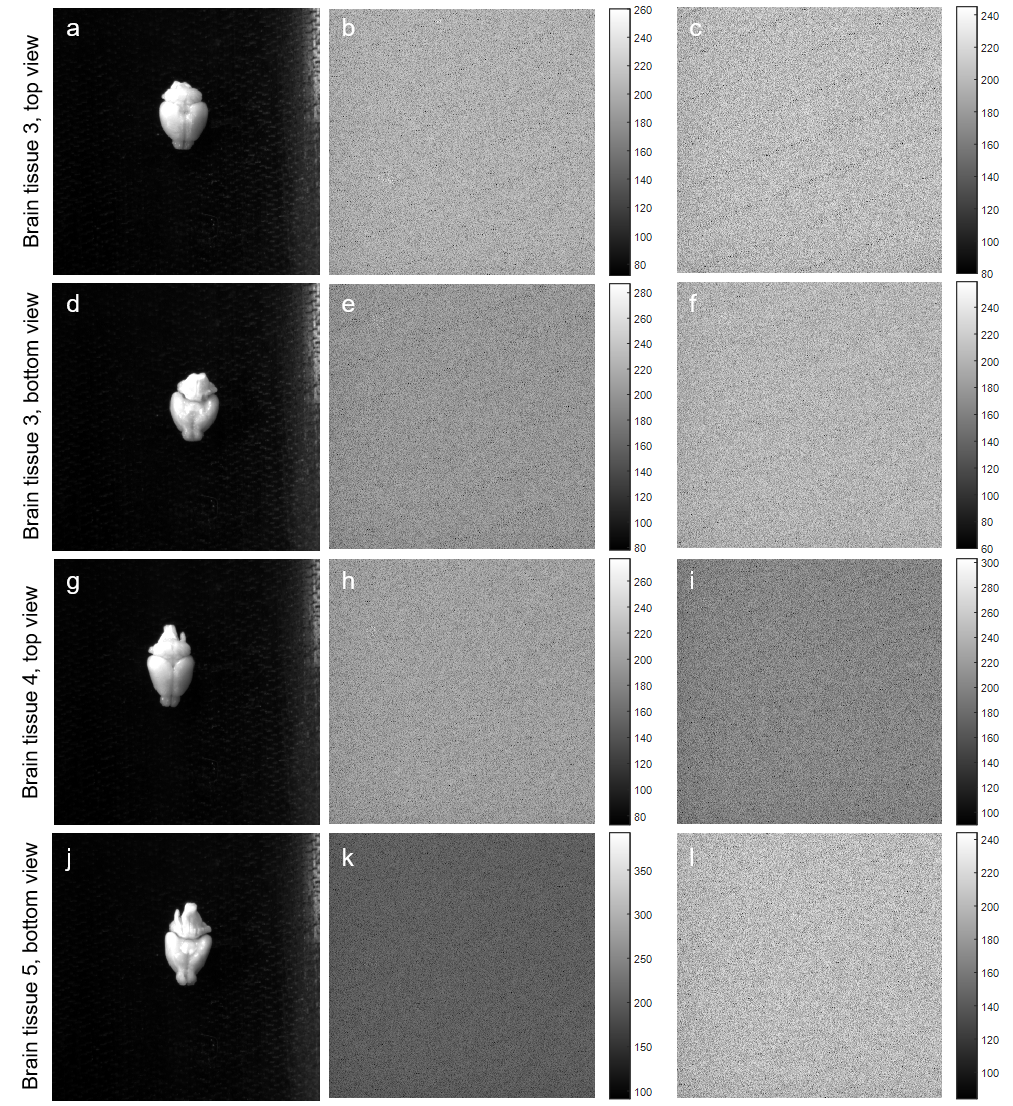


**Figure S10. Fluorescence test of intact brain tissues.** Positioning photograph of the **(a)** top view and **(d)** bottom view of brain tissue 3 (eNpHR 3.0(+), Y_2_O_3_: Eu(+)) under white light. Photograph of the **(b)** top view and **(e)** bottom view of brain tissue 3 (fluorescence background) in a darkroom. Photograph of the **(c)** top view and **(f)** bottom view of brain tissue 3 (radioluminescence) under X-ray irradiation. Photographs of brain tissue 4 (eNpHR 3.0(+), Y_2_O_3_: Eu(-)) were displayed in **(g-l)**, with the same layout as **(a-f)**.


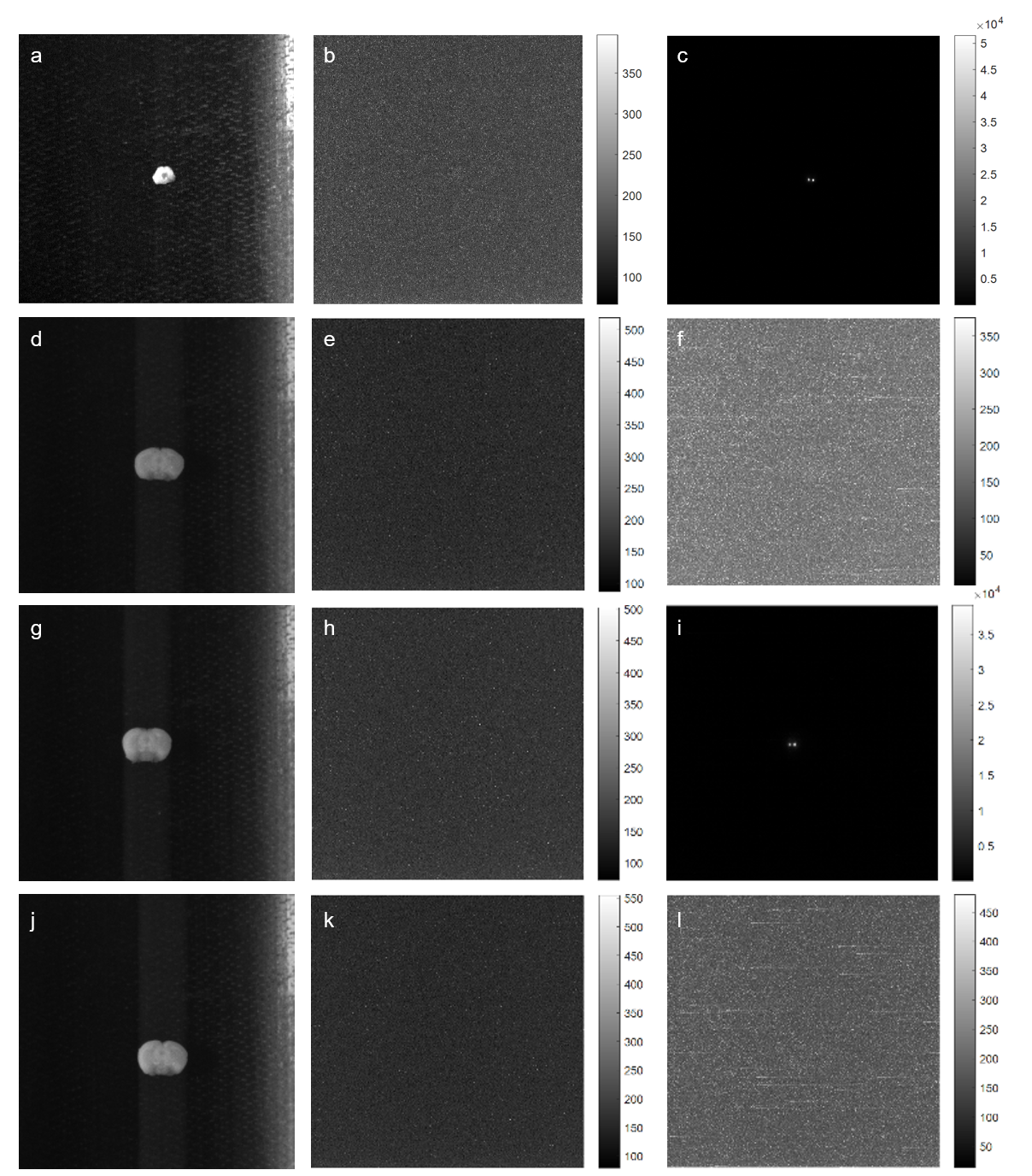


**Figure S11. Fluorescence test of coronal brain sections.** **(a)** Positioning photograph of the coronal section 1 (ArchT (+), Gd_2_O_2_S: Tb (+)) under white light. Photograph of the coronal section 1 **(b)** (fluorescence background) in a darkroom, **(c)** (radioluminescence) under X-ray irradiation. **(d-f)**, **(g-i)** and **(j-l)** respectively show images of the brain section 2 (ArchT (+), Gd_2_O_2_S: Tb (-)), brain section 3 (eNpHR 3.0 (+), Y_2_O_3_: Eu (+)), and brain section 4 (eNpHR 3.0 (+), Y_2_O_3_: Eu (-)), under white liht, in a darkroom, and under X-ray irradiation, with the same layout as **(a-c)**.


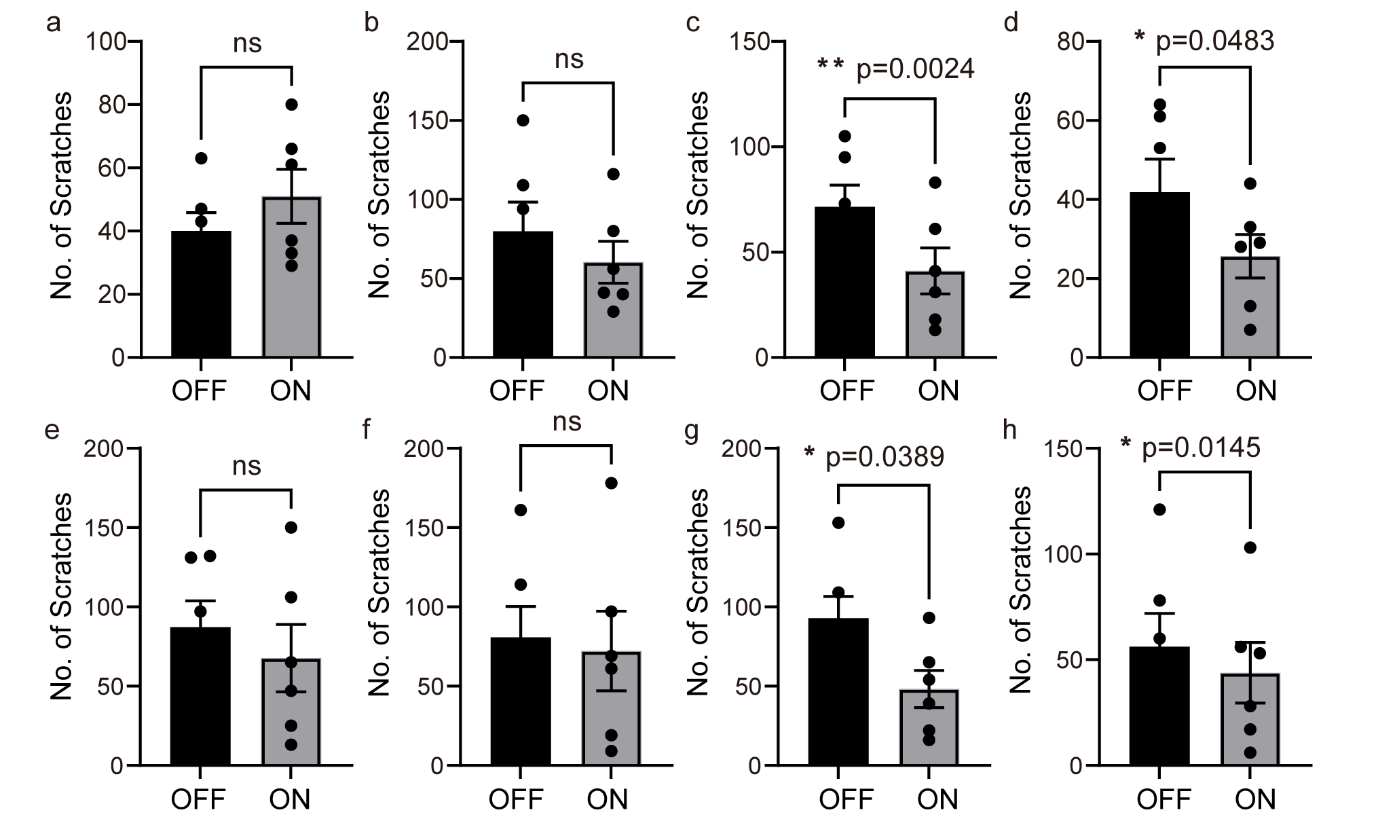


**Figure 12. The effect of different radiation doses on scratching behavior. (a-d)** Mice were injected with ArchT and Gd_2_O_2_S: Tb. The irradiation doses were set of **(a)** 0.57 Gy, **(b)** 1.72 Gy, **(c)** 2.78 Gy, and **(d)** 4.02 Gy, respectively. **(e-h)** Mice were injected with eNpHR 3.0 and Y_2_O_3_: Eu. The irradiation doses were set of **(e)** 0.57 Gy, **(f)** 1.72 Gy, **(g)** 2.78 Gy, and **(h)** 4.02 Gy, respectively.
